# Step In, Step Out from the First Lockdown: An Exploration of COVID-19 Perceptions in France and Quebec

**DOI:** 10.3390/idr13040089

**Published:** 2021-11-22

**Authors:** Jean-Charles David, Kévin Nadarajah, Anta Niang, Sylvain Delouvée, Martin Goyette, Stéphanie Bordel, Alain Somat

**Affiliations:** 1 Department of Psychology, Université Rennes 2, LP3C-EA 1285, F-35000 Rennes, France; jean-charles.david@univ-rennes2.fr (J.-C.D.); kevin.nadarajah@univ-rennes2.fr (K.N.); alain.somat@univ-rennes2.fr (A.S.); 2 Institut Universitaire de Première Ligne en Santé et Services Sociaux (IUPLSSS) du CIUSSS de l’Estrie-CHUS, Sherbrooke, QC J1H 4C4, Canada; anta.niang.ciussse-chus@ssss.gouv.qc.ca; 3 École Nationale d’Administration Publique (ENAP), Montreal, QC H2T 3E5, Canada; martin.goyette@enap.ca; 4 Centre d’Études et d’Expertise sur les Risques, l’Environnement, la Mobilité et l’Aménagement, F-22000 Saint-Brieuc, France; stephanie.bordel@cerema.fr

**Keywords:** COVID-19, perceptions, cross-cultural study, preventive health behaviors

## Abstract

Objective. The objective of this research was to describe and analyze the role of psychological and behavioral factors on perceptions of COVID-19 in France and Quebec at three different times during the pandemic. Design. We conducted three qualitative and quantitative studies (Study 1 *N* = 255, Study 2 *N* = 230, Study 3 *N* = 143). Participants were asked to evaluate psychological and behavioral measures: at the beginning of lockdown (Study 1), during lockdown (Study 2), and during lockdown exit (Study 3). Results. Results of Study 1 show that perceptions of COVID-19 are organized around fear and a sense of threat. During the lockdown, participants mentioned for the first time the health practices to prevent the spread of COVID-19 (Study 2). Psychological and social impacts constitute a central theme in participants’ discourse (Study 2 and 3). Conclusions. The results show that perceptions of risk during a pandemic are socially constructed. Perceptions seem to be influenced by the political and health management of a territory and by the evolution of behavioral and psychological responses.

## 1. Introduction

In December 2019, coronavirus was first identified in Wuhan, China. Since then, many governments have implemented health measures that have proven to be effective in reducing the spread of the virus [1]. Despite the deployment of these measures, populations are facing health [2], economic [3], social [4], cultural [5], environmental [6], and political [7] consequences.

In this context, many studies have been conducted in a wide range of fields to better understand the impacts of the pandemic [8]. They have also helped to provide government authorities with appropriate social and macroeconomic policy responses [9]. In the social sciences particularly, Van Bavel et al. [10] have even called on the scientific community to pursue several research topics related to COVID-19 (e.g., social and cultural context). Thus, several studies have focused on the psychological impacts of the COVID-19 pandemic [11], lockdown [12], and social distancing [13]. These studies have already provided knowledge to help populations adopt the recommendations of epidemiologists and public health experts [14].

### 1.1. Perception of Risk in the Pandemic Context

First, epidemics have marked human history and have caused panic and anxiety among populations [15]. Some studies have shown that the adoption of health practices to control infection was predicted by individuals’ perception of epidemic risk and their level of anxiety [16]. More generally, epidemics provoke strong emotional reactions such as fear. As this was the case for the severe acute respiratory syndrome (SARS) epidemic. The results of a study conducted by Chang [17] strongly suggest that fear of SARS significantly influenced people’s behavior in seeking medical care and that this fear seriously affected access to quality care. Negative emotions and cognitive evaluations are in fact the result of the defensive mechanisms developed by individuals to cope with the threat they perceive [18]. These defense mechanisms can thus be studied and understood using perceptions [19]. They would also be a key to understanding how to properly manage future epidemics [20].

Second, as Slovic argues, “Danger is real, but risk is socially constructed” (p. 689, [21]). Indeed, for many authors, and in particular for the human and social sciences, risk is socially constructed and depends on the perceptions of actors, i.e., the meanings and values they bring into play when they perceive situations [22,23]. Research on collective risks (related to ecosystem, health, natural, technological and food risks) and the crises they provoke shows that there is a common and socially shared knowledge on the subject, which, according to Gilbert [24], is linked to the fact that these risks can threaten the integrity and interests of the populations concerned. Furthermore, Rudnick [25] argues that the international dynamics associated with the pandemic are important because societies are measured by how they learn from these collective crises. Thus, in order to understand these collective risks and the impact of prevention measures on them, more and more researchers are looking at their social perceptions.

Finally, because ‘risk is intrinsically embedded in time’ (p. 530, [26]), it seems essential to put these different research topics into perspective with the pandemic’s temporal dimension. This temporal dynamic can be shaped by governmental decisions or by evolution in media discourse. These can shape the relationship of individuals to a virus, as was the case with SARS in 2003 [27] and more recently with COVID-19 [28]. Political actions can also generate excessive behavior, or blind compliance, as well as variations in emotional levels and sensitivity to social risks [29]. Time dynamics related to measures of physical and social distancing can also highlight socially supportive behaviors as a lever of resilience, which is one of the possible long-term effects of the epidemic [30], with respect to perceived loneliness [31].

The perception of health risks has therefore been widely studied in recent decades, notably during the COVID-19 pandemic [32]. However, to our knowledge, few studies have focused on how psychosociological, cultural, and temporal factors structure the way populations make sense of emerging health threats.

### 1.2. The Present Study

**Figure 1:**
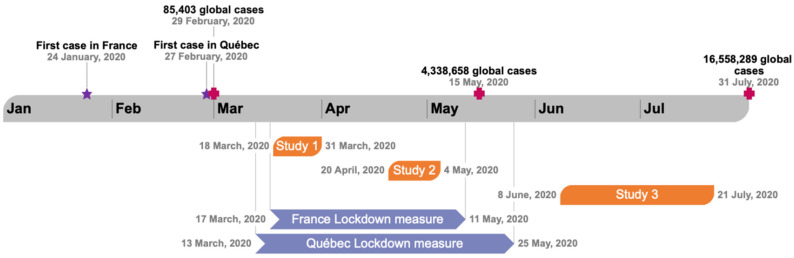
Survey timeline. Quebec adopted the first measures to ease the lockdown on 4 May 2020. Only the Grand Montréal has delayed the measures until 25 May 2020.

The objective of this research was to describe and analyze the role of psychological and behavioral factors on perceptions of COVID-19 in France and Quebec at three distinct times during the pandemic—i.e., at the beginning of lockdown (Study 1), at 1 month of lockdown (Study 2), and at the lockdown exit (≥1 month) (Study 3)—in France and Quebec, and the individuals’ responses towards COVID-19 (Figure 1 show the survey timeline).

In an exploratory perspective, we chose to compare two territories with different political systems, a Western European country, France, and a North American country, Canada. France is a unitary republic whereas Canada is a monarchy with a federated parliamentary system. This implies that the health measures for COVID-19 in France are the same for the whole country, whereas the responses to the epidemic are made by each province in Canada. In order to control the political and health decisions made across Canada on the perception of the virus, we chose to focus on one province, Quebec, where the implementation of the lockdown was relatively similar to France (e.g., the start of the lockdown). The choice of these two territories also allows us to bring a comparative dimension, as they share a common history [33] and have the same official language, French. Despite these similarities, cross-cultural differences remain and can have an impact on the perception of the epidemic [34]. Indeed, Guan et al. [35] highlight the influence of national culture (i.e., the shared meanings and practices within a nation) on collective actions and norms (macroeconomic, political, respect for barrier gestures) during the COVID-19 pandemic. We were thus able to compare two territories with common characteristics, and having put in place sanitary measures (e.g., barrier gestures; Lockdown) at relatively similar periods but culturally and politically distant, which may influence the perception of the pandemic.

## 2. Study 1, COVID-19 Perceptions at the Beginning of Lockdown

### 2.1. Methods

#### 2.1.1. Procedure

Participants aged 18 or older were recruited to participate in the study at the start of the lockdown (from 18 to 31 March). The lockdown and therefore mobility restrictions did not allow us to use traditional means to recruit participants. We have therefore carried out a survey on Limesurvey^®^. We chose to disseminate the survey link on open access group pages of the 3 largest social media platforms (Linkedin^®^; Facebook^®^ and Twitter^®^). This allowed us to reach people where they were, as these social media sites saw an increase in usage during the lockdown [36]. To prevent individuals from completing the questionnaire multiple times, only one questionnaire could be submitted from a particular IP address.

#### 2.1.2. Participants

In total, 486 people opened the survey links, and 255 completed it (a response rate of 52.4%). Our sample includes 203 women, aged from 18 to 77 years (M = 37, SD = 12.8). A total of 146 resided in France and 109 in Quebec (Table 1).

#### 2.1.3. Measures

*Perceptions of the virus*. These perceptions were measured by a free association task [37]. This method is based on free association and consists of a prime word (here “coronavirus”) to which participants have to associate spontaneously four words, phrases or feelings.

*Health practices*. Each participant evaluated “barrier gestures” that were advocated by WHO such as regular hand washing or monitoring for symptoms of infection, on a scale from 1 (not at all) to 5 (absolutely). An average compliance score was produced for all participants (α= 0.59).

*Personal involvement*. We used the three components proposed by Ernst-Vintila et al. [38]. Participants completed a scale adapted from previous work [39] to assess personal involvement in the Coronavirus. Exploratory factor analysis with Oblimin rotation revealed a three-factor structure yielding a satisfactory fit. The first factor referred to “personal identification” (e.g., “I feel particularly concerned by Coronavirus”). The second factor referred to the valuation of the object (e.g., “I think that coronavirus issues are important in our society“). The third factor referred to the perceived capacity for action (e.g., “I feel that through my thoughts and knowledge of coronavirus issues, I am in a position to really act.”). Maximum involvement occurs when a person feels concerned about the virus (ID+), when issues related to the virus are considered important (VAL+), and when something can address it (PCA+). McDonald’s omega coefficient was 0.80 indicating good test reliability.

#### 2.1.4. Data Analysis

We conducted a correspondence factor analysis on the participants’ evocations with a frequency greater than or equal to 6 (see [40]). We studied the relationship of these evocations with several variables: the location of residence (Quebec, France), the individual’s identification with the object, valuation of the object, perceived capacity to act and health practices. For each measure, “low” and “high” classes were defined with a median split.

We also used univariate ANOVAs to examine whether there was a difference between the French and Quebecers in their identification, valuation, perceived capacity for action towards COVID-19, and their health practices.

### 2.2. Results

#### 2.2.1. Perceptions of COVID-19 at the Beginning of Lockdown

**Figure 2:**
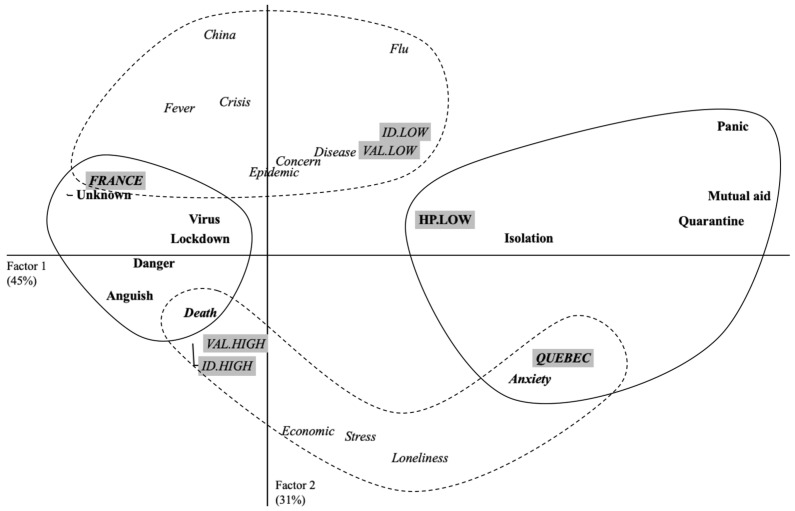
Graphic representation of results provided by correspondence factor analysis considering Factor 1 and Factor 2 for study 1 (beginning of Lockdown).

The CORR. F. A. highlights two factors that explain 75.68% of the table’s inertia (Factor 1 = 44.96%; Factor 2 = 30.72%). Factor 1 receives a contribution from the terms of the variable “Territories”: CF (France) = 0.26 + CF (Quebec) = 0.36, and “health practices”: CF (low health practices) = 0.11, i.e., a contribution of 73%, to the formation of the factor. Factor 2 receives a contribution from the terms of the variable “Territories”: CF (France) = 0.10 + CF (Quebec) = 0.14, and “personal involvement”: CF (high identification) = 0.14, + CF (low identification) = 0.20, + CF (high valuation) = 0.13, + CF (low valuation) = 0.16, i.e., a contribution of 87%, to the formation of the factor. Figure 2 shows this configuration.

Factor 1 shows that the perceptions of French participants differ from those of Quebecers. The French associated coronavirus with *danger*, *anguish*, or *death*. The health measures deployed in France during the first study are also described by the French as *lockdown*. For Quebecers participants, the coronavirus is more associated with mental health by the evocation of terms such *anxiety*. Factor 1 also shows that health practices such as hand washing or greeting without shaking hands determined participant’s evocations. Participants who least adopted these practices used negative terms such as *panic*, *isolation*, and *quarantine* to describe the coronavirus. Finally, the term *mutual aid* is evoked by these same participants for the importance of social ties during the period of lockdown.

Factor 2 shows another difference between territories. The French participants associated the coronavirus with *fever*, i.e., the infectious nature of the coronavirus. They also referred to the geographical origin of the coronavirus with the evocation of *China*. The economic and social consequences of lockdown were more important in the discourse of Quebecers. Factor 2 also shows a link between the participant’s personal involvement and their evocations. For example, the participants least involved with the virus produced a particularly descriptive discourse with evocations such as *disease* and also associated coronavirus with *flu*.

#### 2.2.2. Personal Involvement and Health Practices

Results (Table 1) show that Quebecers identified more with the virus (F (1, 253) = 5.71, *p* = 0.018, *η*^2^ = 0.022), placed more importance on COVID-19 issues (F (1, 253) = 6.64, *p* = 0.011, *η*^2^ = 0.026), and perceived more capacity to act on the pandemic (F (1, 253) = 41, *p* < 0.001; *η*^2^ = 0.139). No significant differences were observed between the two populations regarding their compliance to health practices (*p* = 0.992).

## 3. Study 2, COVID-19 Perceptions during the Lockdown

### 3.1. Method

#### 3.1.1. Procedure

We used the same procedure as in Study 1, except that the participants were recruited 1 month after the start of the first lockdown (from 20 April to 4 May). Participants were also exposed to the Study 1 measures, but they also had to answer an additional question regarding their attitudes towards lockdown measures.

#### 3.1.2. Participants

In total, 1131 people opened the survey links, and 230 completed it (a response rate of 20.3%). Our includes 172 women, aged from 18 to 83 years (M = 36.5, SD = 13.6). A total of 119 resided in France and 111 in the province of Quebec (Table 2).

#### 3.1.3. Measures

The items to measure perceptions of virus, health practices (α = 0.59), and personal involvement (ω = 0.78) were the same as in Study 1.

*Attitudes towards lockdown measures*. Attitude is the “evaluation” of a subject or group with respect to an object, an action, another individual or group [41]. To measure participants’ attitudes towards lockdown measures, they assessed their global attitude (“Overall, in relation to lockdown, I am …“) on a single-item scale from 1 (totally against) to 7 (totally in favor) adapted from the favorability item proposed by Fabrigar, Krosnick, and MacDougall [42].

### 3.2. Results

#### 3.2.1. Perceptions of COVID-19, 1 Month after the Beginning of Lockdown

**Figure 3:**
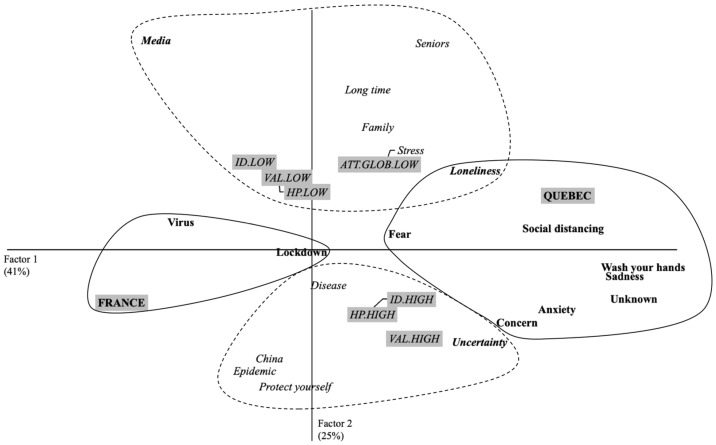
Graphic representation of results provided by correspondence factor analysis considering Factor 1 and Factor 2 for study 2 (1 month of Lockdown).

As in Study 1, a correspondence factor analysis was used on the participants’ evocations with a frequency greater than or equal to 5. The CORR. F. A. highlights two factors that explain 65.91% of the table’s inertia (Factor 1 = 44.17%; Factor 2 = 24.74%). Factor 1 receives a contribution from the terms of the variable “Territories”: CF (France) = 0.40 + CF (Quebec) = 0.40, i.e., a contribution of 80%, to the formation of the factor. Factor 2 receives a contribution from the terms of the variable “health practices”: CF (low health practices) = 0.10, “personal involvement”: CF (high identification) = 0.10, + CF (low identification) = 13, + CF (high valuation) = 0.15, + CF (low valuation = 0.12, and “attitude toward lockdown measures”: CF (low attitude toward lockdown measures) = 0.11, i.e., a contribution of 71%, to the formation of the factor. Figure 3 shows this configuration.

Factor 1 shows that French participants still had differences in their perceptions compared to Quebecers. French participants associated coronavirus with *virus* and *lockdown.* On the contrary, Quebecer participants were more specific in their evocations, using terms such as *social distancing* and *washing your hands*. Moreover, they used words such as *fear*, *concern*, and *anxiety*.

Factor 2 indicates that personal involvement, health practices, and attitudes towards lockdown measures emerged from the participants’ discourse. For example, people who had low scores on these dimensions evoked ideas of constraint such as *long term* or *stress* and social separation such as *seniors* or *family*. By contrast, participants who had high scores in terms of personal involvement and health practice used terms such as *epidemic* or *protecting yourself*. 

#### 3.2.2. Personal Involvement, Health Practices and Attitudes toward Lockdown Measures

Results show that Quebecers (compared to French) identified more with the virus (*F* (1, 228) = 4.24, *p* = 0.041, *η*^2^ = 0.018), placed more importance on COVID-19 issues (*F* (1, 228) = 4.77, *p* = 0.030, *η*^2^ = 0.020), and perceived more capacity to act on the pandemic (*F* (1, 228) = 20.05, *p* < 0.001, *η*^2^ = 0.083). Compared to the French, Quebecers agreed more with the lockdown measures deployed on their territory (*F* (1, 228) = 4.19, *p* = 0.042, *η*^2^ = 0.018). Compliance toward health practices were not significantly different between the two populations (*F* (1, 228) = 1.68, *p* = 0.197, *η*^2^ = 0.007).

## 4. Study 3, COVID-19 Perceptions at the Lockdown Exit

### 4.1. Method

#### 4.1.1. Procedure

We used the same procedure as in Study 1, but this time the participants were recruited at the lockdown exit (from 8 June to 20 July). Participants were also exposed to the same measures as in Study 1, except that they had to answer an additional question related to their attitudes towards lockdown exit measures.

#### 4.1.2. Participants

In total, 1416 people opened the survey links, and 143 completed it (a response rate of 10.1%). Our sample includes 108 women, aged from 19 to 76 years (M = 35.9, SD = 12.8). 71 resided in France and 72 in Quebec (Table 3).

#### 4.1.3. Measures

The items used to measure perceptions of virus, health practices (α = 0.74), and personal involvement (ω = 0.86) were the same than in Study 1 and 2.

*Attitudes towards lockdown exit measures*. The same method as in Study 2 was used to develop the attitude item. The only difference is the object of attitude assessed by the participants. Indeed, they had to assess their global attitude towards lockdown exit measures (“Overall, in relation to lockdown exit, I am …”) on a single-item scale from 1 (totally against) to 7 (totally in favor).

### 4.2. Results

#### 4.2.1. Perceptions of COVID-19 at the Lockdown Exit

**Figure 4:**
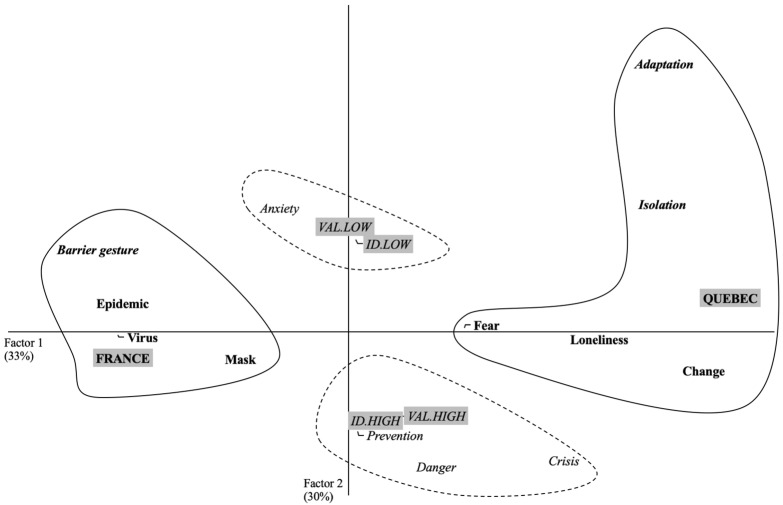
Graphic representation of results provided by correspondence factor analysis considering Factor 1 and Factor 2 for study 3 (Lockdown exit).

We conducted a third correspondence factor analysis on the participants’ evocations with a frequency greater than or equal to 5. The CORR. F. A. highlights two factors that explain 63.15% of the table’s inertia (Factor 1 = 33.09%; Factor 2 = 30.06%). Factor 1 receives a contribution from the terms of the variable “Territories”: CF (France) = 0.37 + CF (Quebec) = 0.49, i.e., a contribution of 86%, to the formation of the factor. Factor 2 receives a contribution from the terms of the variable “personal involvement”: CF (high identification) = 0.18, + CF (low identification) = 0.19, + CF (high valuation) = 0.18, + CF (low valuation) = 0.22, i.e., a contribution of 77%, to the formation of the factor. Figure 4 shows this configuration.

Factor 1 shows the same pattern as in the previous study, in terms of differences between French and Quebecers evocations. As in Study 2, the French participants associated very descriptive terms with coronavirus, such as *virus* or *epidemic*. However, during this phase, these participants mentioned terms related to health practices such as *barrier gesture* and *mask*. While the discourse of Quebecers is still characterized by *fear*, *loneliness*, and *isolation*, these participants also mentioned ideas about resilience by referring to terms such as *adaptation* and *change*.

Factor 2, on the other hand, reveals a contradiction between participants with high and low levels of involvement. Highly involved participants reported the importance of barrier gestures with evocations such as *danger* and *prevention*. They also discussed the long-term consequences of the virus with the term *crisis*. Finally, participants with low levels of involvement used the term *anxiety* to refer to the psychological state of individuals during the pandemic.

#### 4.2.2. Personal Involvement, Health Practices, and Attitudes toward Lockdown Exit Measures

Results show no significant differences between French and Quebecers regarding their identification with COVID-19 (*F* (1, 141) = 0.227, *p* = 0.635, *η*^2^ = 0.002), their valuation of the COVID-19 (*F* (1, 141) = 0.546, *p* = 0.461, *η*^2^ = 0.004), and their attitudes toward lockdown exit measures (*F* (1, 141) = 0.374, *p* = 0.542, *η*^2^ = 0.003). Nevertheless, Quebecers perceived more capacity to act on the pandemic (*F* (1, 141) = 3.19, *p* = 0.076, *η*^2^ = 0.022). In addition, they were more compliant with Health practices (*F* (1, 141) = 7.63, *p* = 0.007, *η*^2^ = 0.051).

## 5. General Discussion

The objective of this research was to describe and analyze the role of psychological and behavioral factors on perceptions of COVID-19 in France and Quebec at three different times during the pandemic. In line with our objectives, the results of these studies show how people structured their perceptions of COVID-19 during the first lockdown. For example, the way people interpret emerging health threats might depend on the policy and health measures in place in a territory. Indeed, at the beginning of the study, the perceptions of the Quebecers and the French were more focused on their feelings (Study 1), and fear in particular. The feeling of fear has also been an important characteristic of previous epidemics (e.g., SARS) [17], and our results seem to confirm this phenomenon. Indeed, the unprecedented nature of the COVID-19 pandemic confronts populations with the unknown, loss of control, and feelings of powerlessness [43]. As a result, the epidemic generates negative emotions such as fear and anxiety; fear is therefore a normal reaction to danger that helps guide our choices and actions by avoiding excessive risks to our health [44]. Fear is a fundamental adaptive defense mechanism for survival, and involves several biological processes in preparing a response to perceived threatening events [45]. However, the results of these studies raise questions about the way people conceptualize changes over time. Indeed, if the participants’ perceptions were strongly marked by emotions during the first study, it seems that they then progressively de-centered themselves from their emotions. Thus, they mentioned the lockdown measures (Study 2), and then the time of lockdown seems to have allowed them to mention more elements related to the individual management (e.g., barrier gesture) of the COVID-19 (Study 3). These results could suggest that individuals had tempered their strong emotions by managing the risk more analytically [46].

Furthermore, these results are in line with work that has identified socio-cultural [47] and emotional [48] factors important for effective risk communication. Indeed, these studies suggest that individuals’ perceptions and adoption of preventive behaviors are not simply associated with a set of medical or scientific information but also with a given time period and cultural characteristics. Thus, these studies draw attention to the role of perceptions in pandemic management, as already advocated by Van Bavel et al. [10] at the beginning of pandemics. The results of these studies show to policy makers that relatively similar communication strategies can have different effects depending on the psycho-sociological, behavioral, and cultural anchors of the individuals.

However, these studies have some limitations. Firstly, the small sample size is one of the limits of these studies. This does not allow us to cover the wide diversity of perceptions that can exist within a territory. In addition, a larger sample could have allowed us to explore perceptions within each territory in order to assess the existence of differences (e.g., between town and country). Secondly, our method does not allow us to study “changes” over time. To do so, it would have been necessary to carry out repeated measurements on the same individuals. Nevertheless, our aim was not to study changes in perceptions of COVID-19, but rather to explore these perceptions in order to shed light on how individuals’ perceptions may be structured, depending on the territory where they live. Future longitudinal research could study the evolution of individuals’ psychological and behavioral responses to political and health decisions implemented in a territory. This research should also take into account the socio-demographic characteristics of the participants, as they may influence the perception of health risks (e.g., [49]).

Finally, if these studies suggest that the lockdown period was, for many, a time for reflection, introspection, and taking a step back from COVID-19, the negative impacts of the pandemic, particularly on the mental health of many people, should not be overlooked.

## 6. Conclusions

Epidemic management is often conceptualized by policy makers through the notion of risk. Indeed, they measure risk as the probability of contracting a disease multiplied by the magnitude of its consequences [50]. However, these three studies suggest that perceptions of COVID-19 risk semantics may also be influenced by complex psychological, social and political processes. Faced with an epidemic threat such as COVID-19, it would be necessary to consider the evolution of people’s perceptions in order to adapt communications and effectively mobilize experiential (official messages from the authorities at the beginning of a crisis) and/or rational (communication of epidemiological data during the epidemic) content. In addition to communication devices, it would be wise to restructure the environment (e.g., the nudge theory) in order to guide populations towards health behaviors that promote public health. Far from a representation that would oppose emotion against reason, this study suggests the development of prevention measures that properly integrate the study of perceptions to support the management of epidemics.

## Figures and Tables

**Table 1:** Characteristics of the participants, means, and standard deviations for each variable (study 1).

Variables	Overall (*n* = 255)	France (*n* = 146)	Quebec (*n* = 109)
Age in year	37 ± 12.8	36.1 ± 12.8	38.2 ± 12.7
Gender			
Men	49	27	22
Women	203	117	86
Other	3	2	1
Social professional category			
Managers and senior professionals	73	46	27
Employees	63	37	26
Students	45	33	12
Intermediate Professions	21	7	14
Persons without professional activity	9	6	3
Retired	17	7	10
Liberal Professions	3	3	0
Workers	4	3	1
Farmers	1	1	0
Other	19	3	16
Identification with COVID-19	4.50 ± 0.68	4.41 ± 0.75	4.61 ± 0.56
Valuation of the COVID-19	4.58 ± 0.51	4.50 ± 0.58	4.67 ± 0.38
Perceived capacity for action	3.61 ± 0.78	3.36 ± 0.70	3.94 ± 0.76
Health practices	4.15 ± 0.45	4.15 ± 0.48	4.15 ± 0.40

**Table 2:** Characteristics of the participants, means, and standard deviations for each variable (study 2).

Variables	Overall (*n* = 230)	France (*n* = 119)	Quebec (*n* = 111)
Age in year	36.5 ± 13.6	31.7 ± 12.2	41.6 ± 13.1
Gender			
Men	55	34	21
Women	172	84	88
Other	3	1	2
Social professional category			
Managers and senior professionals	75	39	36
Employees	52	24	28
Students	51	36	15
Intermediate Professions	18	6	12
Persons without professional activity	8	7	1
Retired	14	3	11
Liberal Professions	5	1	4
Workers	1	1	0
Farmers	2	2	0
Other	4	0	4
Identification with COVID-19	4.33 ± 0.71	4.24 ± 0.74	4.43 ± 0.67
Valuation of the COVID-19	4.45 ± 0.57	4.37 ± 0.59	4.53 ± 0.52
Perceived capacity for action	3.52 ± 0.81	3.30 ± 0.80	3.76 ± 0.75
Health practices	4.12 ± 0.48	4.08 ± 0.46	4.16 ± 0.50
Attitudes toward lockdown measures	4.22 ± 0.93	4.10 ± 0.98	4.35 ± 0.87

**Table 3:** Characteristics of the participants, means, and standard deviations for each variable (study 3).

Variables	Overall (*n* = 143)	France (*n* = 72)	Quebec (*n* = 71)
Age in year	35.9 ± 12.8	31.8 ± 10.6	40.1 ± 13.5
Gender			
Men	33	16	17
Women	108	55	53
Other	2	1	1
Social professional category			
Managers and senior professionals	45	25	20
Employees	28	15	13
Students	30	19	11
Intermediate Professions	8	5	3
Persons without professional activity	8	2	6
Retired	10	2	8
Liberal Professions	12	2	10
Workers	0	0	0
Farmers	1	1	0
Other	1	1	0
Identification with COVID-19	4.18 ± 0.85	4.15 ± 0.85	4.21 ± 0.85
Valuation of the COVID-19	4.26 ± 0.76	4.21 ± 0.76	4.31 ± 0.77
Perceived capacity for action	3.51 ± 0.79	3.39 ± 0.80	3.63 ± 0.78
Health practices	3.90 ± 0.62	3.76 ± 0.62	4.04 ± 0.59
Attitudes toward lockdown measures	3.67 ± 0.91	3.72 ± 0.88	3.63 ± 0.94

## Data Availability

Data from this study are openly available at https://osf.io/u3wjv (accessed on 22 October 2021).

## References

[1] Fang Y., Nie Y., Penny M. (2020). Transmission dynamics of the COVID-19 outbreak and effectiveness of government interventions: A data-driven analysis. J. Med. Virol..

[2] Guan W., Liang W., Zhao Y., Liang H., Chen Z., Li Y., Liu X., Chen R., Tang C., Wang T. (2020). Comorbidity and its impact on 1590 patients with COVID-19 in China: A Nationwide Analysis. Respir. Med..

[3] Fernandes N. (2020). Economic Effects of Coronavirus Outbreak (COVID-19) on the World Economy. SSRN Electron. J..

[4] Nicola M., Alsafi Z., Sohrabi C., Kerwan A., Al-Jabir A., Iosifidis C., Agha M., Agha R. (2020). The socio-economic implications of the coronavirus and COVID-19 pandemic: A review. Int. J. Surg..

[5] Bruns D.P., Kraguljac N.V., Bruns T.R. (2020). COVID-19: Facts, Cultural Considerations, and Risk of Stigmatization. J. Transcult. Nurs..

[6] Zambrano-Monserrate M.A., Ruano M.A., Sanchez-Alcalde L. (2020). Indirect effects of COVID-19 on the environment. Sci. Total. Environ..

[7] Bol D., Giani M., Blais A., Loewen P.J. (2020). The effect of COVID-19 lockdowns on political support: Some good news for democracy?. Eur. J. Politi-Res..

[8] Torales J., O’Higgins M., Castaldelli-Maia J.M., Ventriglio A. (2020). The outbreak of COVID-19 coronavirus and its impact on global mental health. Int. J. Soc. Psychiatry.

[9] McKibbin W., Fernando R. (2021). The Global Macroeconomic Impacts of COVID-19: Seven Scenarios. Asian Econ. Pap..

[10] Van Bavel J.J., Baicker K., Boggio P.S., Capraro V., Cichocka A., Cikara M., Crockett M.J., Crum A.J., Douglas K.M., Druckman J.N. (2020). Using social and behavioural science to support COVID-19 pandemic response. Nat. Hum. Behav..

[11] Bonetto E., Delouvée S., Mahfud Y., Adam-Troian J. (2020). National Identification, a Social Cure for COVID-19? Evidence from 67 Countries. PsyArXiv.

[12] Atalan A. (2020). Is the lockdown important to prevent the COVID-19 pandemic? Effects on psychology, environment and economy-perspective. Ann. Med. Surg..

[13] Cerbara L., Ciancimino G., Crescimbene M., La Longa F., Parsi M.R., Tintori A., Palomba R. (2020). A nation-wide survey on emotional and psychological impacts of COVID-19 social distancing. Eur. Rev. Med Pharmacol. Sci..

[14] Pennycook G., McPhetres J., Zhang Y., Lu J.G., Rand D.G. (2020). Fighting COVID-19 Misinformation on Social Media: Experimental Evidence for a Scalable Accuracy-Nudge Intervention. Psychol. Sci..

[15] Gonsalves G., Staley P. (2014). Panic, Paranoia, and Public Health—The AIDS Epidemic’s Lessons for Ebola. N. Engl. J. Med..

[16] Wang C., Pan R., Wan X., Tan Y., Xu L., Ho C.S., Ho R.C. (2020). Immediate Psychological Responses and Associated Factors during the Initial Stage of the 2019 Coronavirus Disease (COVID-19) Epidemic among the General Population in China. Int. J. Environ. Res. Public Health.

[17] Chang H.-J., Huang N., Lee C.-H., Hsu Y.-J., Hsieh C.-J., Chou Y.-J. (2004). The Impact of the SARS Epidemic on the Utilization of Medical Services: SARS and the Fear of SARS. Am. J. Public Heal..

[18] Emobbs D., Hagan C., Dalgleish T., Esilston B., Prévost C. (2015). The ecology of human fear: Survival optimization and the nervous system. Front. Neurosci..

[19] Jaspal R., Nerlich B. (2020). Social representations, identity threat, and coping amid COVID-19. Psychol. Trauma Theory Res. Prac. Policy.

[20] Idoiaga Mondragon N., Gil de Montes L., Valencia J. (2017). Understanding an Ebola outbreak: Social representations of emerging infectious diseases. J. Health Psychol..

[21] Slovic P. (1999). Trust, Emotion, Sex, Politics, and Science: Surveying the Risk-Assessment Battlefield. Risk Anal..

[22] Joffe H. (2003). Risk: From perception to social representation. Br. J. Soc. Psychol..

[23] Apostolidis T., Dany L. (2012). Pensée sociale et risques dans le domaine de la santé: Le regard des représentations sociales. Psychol. Française.

[24] Gilbert C. (2003). La fabrique des risques. Cah. Int. Sociol..

[25] Rudnick A. (2020). Social, Psychological, and Philosophical Reflections on Pandemics and Beyond. Societies.

[26] DAS T., Teng B.-S. (2001). Strategic risk behaviour and its temporalities: Between risk propensity and decision context. J. Manag. Stud..

[27] Washer P. (2004). Representations of SARS in the British newspapers. Soc. Sci. Med..

[28] Gao J., Zheng P., Jia Y., Chen H., Mao Y., Chen S., Wang Y., Fu H., Dai J. (2020). Mental health problems and social media exposure during COVID-19 outbreak. PLoS ONE.

[29] Schaller M., Murray D.R., Bangerter A. (2015). Implications of the behavioural immune system for social behaviour and human health in the modern world. Philos. Trans. R. Soc. B Biol. Sci..

[30] Chen S., Bonanno G.A. (2020). Psychological adjustment during the global outbreak of COVID-19: A resilience perspective. Psychol. Trauma Theory Res. Prac. Policy.

[31] Luchetti M., Lee J.H., Aschwanden D., Sesker A., Strickhouser J.E., Terracciano A., Sutin A.R. (2020). The trajectory of loneliness in response to COVID-19. Am. Psychol..

[32] Bhagavathula A.S., Aldhaleei W.A., Rahmani J., Khubchandani J. (2020). Knowledge, Attitude, Perceptions and Practice towards COVID-19: A Systematic Review and Meta-Analysis. Infect. Dis..

[33] France-Canada-Québec (2008). 400 ans de Relations D’exception: 400 ans de Relations D’exception.

[34] Herzlich C., Adam P. (1994). Sociologie de la Maladie et de la Médecine.

[35] Guan Y., Deng H., Zhou X. (2020). Understanding the impact of the COVID-19 pandemic on career development: Insights from cultural psychology. J. Vocat. Behav..

[36] Barhoumi M., Jonchery A., Lombardo P., Le Minez S., Mainaud T., Raynaud E., Pailhé A., Solaz A., Pollak C. (2020). Les inégalités sociales à l’épreuve de la crise sanitaire: Un bilan du premier confinement. France, Portrait Social.

[37] Lo Monaco G., Piermattéo A., Rateau P., Tavani J.L. (2017). Methods for Studying the Structure of Social Representations: A Critical Review and Agenda for Future Research: Methods and Structure of Social Representations. J. Theory Soc. Behav..

[38] Ernst-Vintila A., Delouvée S., Roland-Lévy C. (2011). Under threat. Lay thinking about terrorism and the three-dimensional model of personal involvement: A social psychological analysis. J. Risk Res..

[39] Demarque C., Monaco G.L., Apostolidis T., Guimelli C. (2012). Socialisation, perspectives temporelles et implication personnelle: Une étude dans le champ de l’environnement. Les Cah. Int. Psychol. Soc..

[40] Deschamps J.C., Abric C. (2003). Analyse des correspondances et variations des contenus de représentations sociales. Méthodes D’étude des Représentations Sociales.

[41] Maio G.R., Haddock G. (2009). The Psychology of Attitudes and Attitude Change.

[42] Brock T.C., Green M.C. (2005). Persuasion: Psychological Insights and Perspectives.

[43] Ahorsu D.K., Lin C.-Y., Imani V., Saffari M., Griffiths M.D., Pakpour A.H. (2020). The Fear of COVID-19 Scale: Development and Initial Validation. Int. J. Ment. Health Addict..

[44] Ornell F., Schuch J.B., Sordi A.O., Kessler F.H.P. (2020). “Pandemic fear” and COVID-19: Mental health burden and strategies. Rev. Bras. Psiquiatr..

[45] Pakpour A.H., Griffiths M.D. (2020). The fear of COVID-19 and its role in preventive behaviors. J. Concurr. Disord..

[46] Slovic P., Finucane M.L., Peters E., MacGregor D.G. (2004). Risk as Analysis and Risk as Feelings: Some Thoughts about Affect, Reason, Risk, and Rationality. Risk Anal..

[47] Wardman J. (2014). Sociocultural vectors of effective risk communication. J. Risk Res..

[48] Engdahl E., Lidskog R. (2012). Risk, communication and trust: Towards an emotional understanding of trust. Public Underst. Sci..

[49] Dryhurst S., Schneider C.R., Kerr J., Freeman A.L.J., Recchia G., van der Bles A.M., Spiegelhalter D., van der Linden S. (2020). Risk perceptions of COVID-19 around the world. J. Risk Res..

[50] Papageorge N.W., Zahn M.V., Belot M., Broek-Altenburg E.V.D., Choi S., Jamison J.C., Tripodi E. (2021). Socio-demographic factors associated with self-protecting behavior during the Covid-19 pandemic. J. Popul. Econ..

